# A microscale anisotropic biaxial cell stretching device for applications in mechanobiology

**DOI:** 10.1007/s10529-013-1381-5

**Published:** 2013-10-16

**Authors:** Dominique Tremblay, Sophie Chagnon-Lessard, Maryam Mirzaei, Andrew E. Pelling, Michel Godin

**Affiliations:** 1 Department of Physics, University of Ottawa, 150 Louis-Pasteur, Ottawa, ON K1N 6N5 Canada; 2 Ottawa-Carleton Institute for Biomedical Engineering, University of Ottawa, 161 Louis Pasteur, Ottawa, ON K1N 6N5 Canada; 3 Department of Biology, University of Ottawa, 30 Marie Curie, Ottawa, ON K1N 6N5 Canada; 4 Institute for Science, Society and Policy, University of Ottawa, 55 Laurier Ave. East, 10th Floor, Ottawa, ON K1N 6N5 Canada

**Keywords:** Anisotropic deformation, Cell stretching, Live-cell imaging, Mechanobiology, Microfabrication, Microfluidics

## Abstract

A multi-layered polydimethylsiloxane microfluidic device with an integrated suspended membrane has been fabricated that allows dynamic and multi-axial mechanical deformation and simultaneous live-cell microscopy imaging. The transparent membrane’s strain field can be controlled independently along two orthogonal directions. Human foreskin fibroblasts were immobilized on the membrane’s surface and stretched along two orthogonal directions sequentially while performing live-cell imaging. Cyclic deformation of the cells induced a reversible reorientation perpendicular to the direction of the applied strain. Cells remained viable in the microdevice for several days. As opposed to existing microfluidic or macroscale stretching devices, this device can impose changing, anisotropic and time-varying strain fields in order to more closely mimic the complexities of strains occurring in vivo.

**Electronic supplementary material:**

The online version of this article (doi:10.1007/s10529-013-1381-5) contains supplementary material, which is available to authorized users.

## Introduction

Mechanical forces play an important role in the development, homeostasis and repair of tissues. This is mainly the result of the mechanosensitivity of many biological remodeling processes at the cellular level such as proliferation, migration, differentiation, apoptosis and extracellular matrix synthesis (Ingber 2006). Cell stretching devices have demonstrated their potential to contribute to our fundamental understanding of pathways in cellular mechanotransduction and mechanosensitivity (Huh et al. 2010; Huang and Nguyen 2013; Kim et al. 2012; Moraes et al. 2010, 2013). When integrated on a microfluidic platform, these devices offer significant improvements over their macroscale counterparts (Wang et al. 2010; Huang et al. 2010), mainly given their potential for high throughput processing as well as their ability to be combined with other on-chip functions. Recently, organ-on-a-chip systems have attracted attention by highlighting the ability to better understand the effects of mechanical forces at the cellular level for different organs (Huh et al. 2010; Kim et al. 2012). In vivo, tissue-embedded cells undergo mechanical strains that often vary spatially and temporally. It is the case in vascular tissues where the combination of the local hemodynamic forces (Frydrychowicz et al. 2008) with the anisotropic mechanical properties of vascular tissues (Duprey et al. 2010; Tremblay et al. 2010) exposed endothelial and smooth muscle cells to complex multi-axial and cyclical deformations. Moreover, these strain fields can induce significant sub-cellular, cellular- and multi-cellular remodeling responses in a frequency and magnitude dependent manner (Balachandran et al. 2011; Goldyn et al. 2009; Jungbauer et al. 2008).

Microfluidic stretching devices have been developed to study single cell response to mechanical deformation or to observe multi-culture cell system mimicking organ-level functions under mechanical stimuli. The elegant work by Huh et al. (2010) demonstrated the ability to mimic organ-level functions in a microfabricated stretching device. They were able to uniaxially stretch a co-culture of alveolar epithelial cells and endothelial cells to examine cellular responses to mechanical deformation in a model of the lung. Using a similar device Kim et al. (2012) demonstrated that human intestinal epithelial cells exhibit changes in cell morphology and increased aminopeptidase activity under cyclic uniaxial stretching. Several groups have now integrated microfabricated stretching devices into microfluidic networks in order to allow for high throughput screening. Huang and Nguyen (2013) have integrated microfabricated uniaxial devices in a high throughput platform allowing the investigation of the effect of various uniaxial stretching conditions on cell response within the same experiment. Other systems have used piston-like structures to deform a membrane on which cells are firmly attached to perform high throughput screening. Kamotani et al. (2008) employed microwells with flexible bottom membranes placed over computer-controlled, piezoelectrically actuated pins inducing a broad range of biaxial strain fields in the same microwells. Similar high throughput devices have also been used to monitor the influence of mechanical substrate strain on β-catenin accumulation in the nucleus or myofibroblast differentiation (Moraes et al. 2010, 2013). Taken together, these devices have strongly contributed to the development of a new class of microfabricated devices capable of studying cellular biological processes under mechanical strain.

Although existing devices have clear utility, an area of improvement would be the integration of full and independent biaxial control of the strain field. While idealized strain fields have provided important insights into strain-induced cellular remodelling processes, imposing more complex strain fields in the future would better mimic in vivo cellular systems. In this study, we build upon existing microfluidic stretcher designs and present a complementary device capable of imposing dynamic anisotropic biaxial strains on cells. In addition, our device can also maintain microfluidic control over the introduction of samples and allowing simultaneous imaging by optical microscopy. This device allows the independent and dynamic control of the strain magnitude and waveform frequency (milliseconds to days) in two orthogonal directions during the same stretching experiment, leading to better replication of complex multi-axial cyclic strains common to in vivo systems. We chose human foreskin fibroblast (HFF) cells as a model system for this study as fibroblasts are well known to sense and respond to strain (Wang et al. 2004). We show that the device can maintain cell viability over several days and allows the study of the same group of cells in response to a changing biaxial strain field.

## Materials and methods

### Working principle of the device

**Fig. 1:**
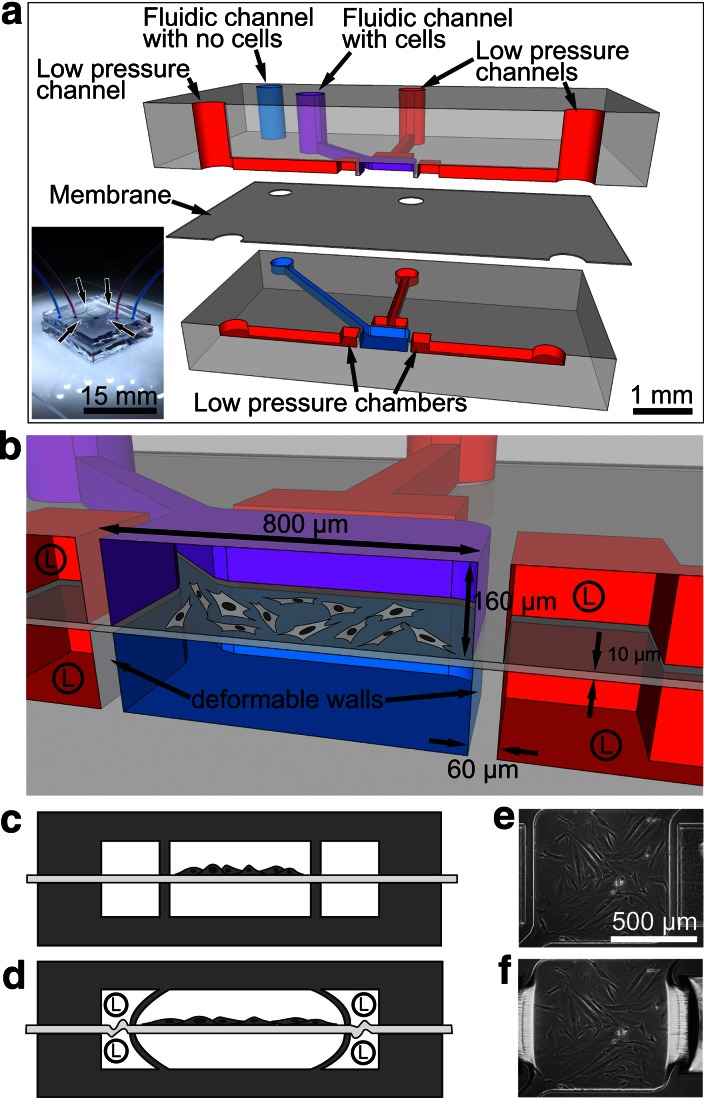
**a** Exploded cross-section of the multi-layer PDMS-based cell stretching device. Low pressure is applied to the low pressure channels (*red*) to induce a deformation in the walls located at each of the four sides of the cell stretching chamber (800 × 800 μm; 10 μm thick membrane). The *top* and *bottom* fluidic channels (*purple* and *blue*) are isolated from each other by a suspended membrane. The *bottom* fluidic channel (*blue*) serves to equilibrate pressures when seeding cells. *Bottom left* of **a**: Photographic image of the assembled device with the *bottom* and *top* fluidic channels (*blue* and *purple* channels) connected and the four low pressure channel inlets (see *arrows*). **b** Detailed view of the assembled device cross-section showing the cell stretching chamber along with the low pressure chambers on both sides (circled “L” indicates low pressure). **c**–**d** Schematic cross-section of the device with cells attached on the membrane and the low pressure chambers under atmospheric pressure conditions (**c**) and low pressure conditions (**d**). **e**–**f** Phase-contrast images of the device viewed from the *top*; two of the four low pressure chambers are visible under atmospheric pressure conditions (**e**) and low pressure conditions (**f**)

We present a microfabricated biaxial stretcher which draws upon the designs presented by Huh et al. (2010) and Huang and Nguyen (2013). Our device is fabricated using poly(dimethylsiloxane) (PDMS; Sylgard184) by multi-layer soft lithography (Fig. 1). Figure 1a shows an exploded cross-section view of the multilayer device with the low pressure and fluidic channels. The 10 μm thick, suspended membrane on which cells adhere and proliferate makes a liquid tight seal between the top fluidic channel (purple) and the bottom fluidic channel (blue). This configuration ensures that no pressure differential is established across the suspended membrane, which prevents any upward or downward displacement of the membrane causing it to stick on the upper or lower surface of the stretching chamber. During the assembly process, the membrane was carefully punctured with a sharp needle to provide access to the channels of the bottom section, while maintaining cleanliness. Also, it was important for the fluidic channels of the top part to be open to the air during the alignment process to equilibrate pressures between the top and bottom fluidic channels, thus avoiding membrane collapse. A detailed fabrication process is included as Supplementary data Fig. 1b shows a cross-section of the device with cells in the stretching chamber. Lateral deformation of the vertical walls occurs when a low pressure is applied (red chambers), which pulls on the attached suspended membrane and induces deformation, as depicted Fig. 1c–f and in Supplementary data: videos A and B. The microfabricated device is maintained on an inverted microscope at 37 °C in a humid atmosphere of 5 % CO_2_/95 % air using a custom incubation chamber in order to perform time-lapse live cell imaging, as described in Supplementary Fig. 1.

### Cell seeding

Before introducing cells, the device’s top and bottom fluidic channels are first wetted and sterilized with 95 % ethanol for 5 min prior to being flushed with autoclaved deionized water for another 5 min. Water is then replaced by a fibronectin solution at 10 μg/ml of HEPES-buffered salt solution (HBSS; 20 mM HEPES at pH 7.4, 120 mM NaCl, 5.3 mM KCl, 0.8 mM MgSO_4_, 1.8 mM CaCl_2_ and 11.1 mM glucose). Once the microfluidic channels are filled with the fibronectin solution, the ends of all tubing leading to the device are placed in a single solution-filled vial. This equilibrates all pressures and completely stops flow within the device, promoting fibronectin functionalization of the membrane. Fibronectin is incubated for 2 h at 5 % (v/v) CO_2_ and 37 °C. Subsequently, the fibronectin solution is replaced with culture medium (DMEM) supplemented with 10 % (v/v) fetal bovine serum and 1 % penicillin/streptomycin at a flowrate of ~10 μl/min. In the mean time, cells cultured in a standard incubator (5 % v/v CO_2_ and 37 °C) are trypsinized and resuspended in culture medium at 2 × 10^6^ cells/ml. The top microchannel is then filled with the culture medium supplemented with cells, whereas the bottom channel is further flushed with fresh culture medium. Individual cells quickly adhere to the fibronectin-coated membrane surface within 10 s under no flow conditions. After 10 s, more cells were carried in the device’s chamber while the cells already present in the chamber remained attached to the membrane. Cells are thus immobilized to the membrane, one by one, until about 70 cells are present in the stretching chamber. Once the cells are adhered to the membrane, flow is again completely stopped by placing all tubing in the same media-filled vial. The cells are left to firmly attach to the fibronectin-coated PDMS membrane overnight. Supplementary Fig. 2 shows the speed at which cells attach to the fibronectin-coated PDMS membrane. The deposited cells are initially somewhat lined up with the fluid flow direction. However, HFFs are very motile and quickly cover the entire surface of the membrane after overnight incubation.

### Image analysis and cell orientation

Cell orientations were quantified using filtered and thresholded phase-contrast images of the cells. A FFT band-pass filter was first applied on the phase-contrast images using ImageJ (http://rsbweb.nih.gov/ij/) to smooth background and isolate cell features. Thresholding was applied to create binary images of the cell features. The orientation of each of the features was computed and record to produce a histogram for each of the stretching conditions.

## Results

### Device performance

**Fig. 2:**
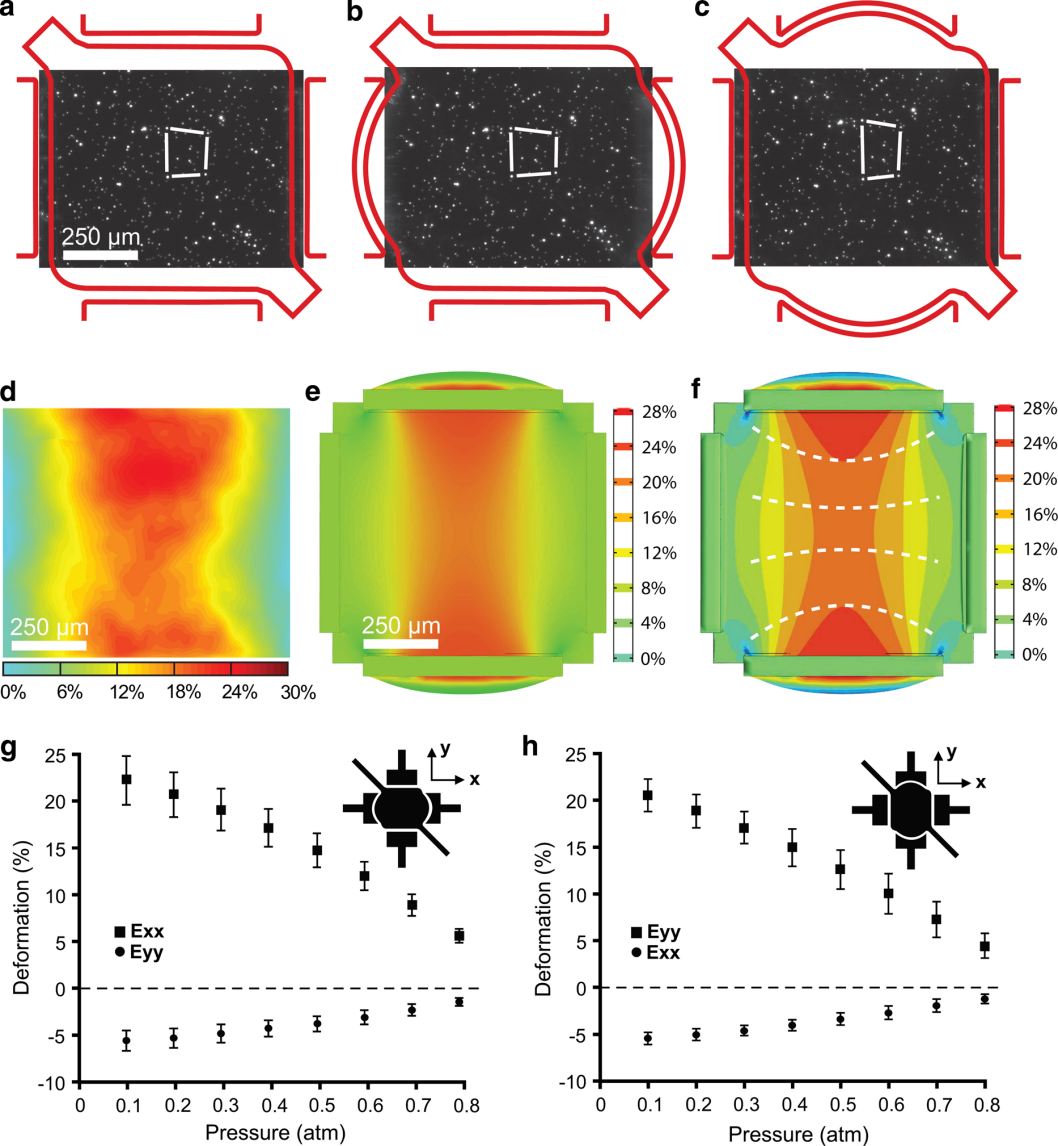
**a**–**c** Fluorescent images showing the fluorescent beads embedded in the membrane, used to monitor membrane deformation. Low pressure chambers are independently activated to induce deformation in the membrane along two *orthogonal* directions. **d** Typical deformation field calculated from the displacements of the embedded beads during uniaxial stretching along the *vertical* direction. **e**–**f** Strain map and contour map of the magnitude of the deformation in the membrane using COMSOL (Burlington, USA). The *white dashed lines* in **f** follows the general alignment of the cells when stretched vertically. **g**–**h** Typical calibration curves illustrating the relationship between pressure and membrane deformation. The symmetry of the devices result in producing very similar calibration curves along the *horizontal* (**g**) and *vertical* direction (**h**)

Prior to performing each stretching experiment, calibration was performed by relating the pressure in the low pressure chambers and the strain field in the flexible membrane. A MATLAB script allowed us to compute the Green strain tensor in the plane of the PDMS membrane by tracking the position of embedded fluorescent beads during stretching. Figure 2a–c illustrates the strain field in the membrane along two orthogonal directions as four embedded particles are tracked (white lines). A strain map can be generated based on the beads tracking computation. Figure 2d, e highlights the agreement between the experimental results and the finite-element simulation of the stretching device in action. While the configuration of the stretching device allowed us to precisely control the strain along the two orthogonal axes, it also leads to a non-uniform strain magnitude over the entire extent of the membrane surface, as depicted in Fig. 2d, f. Representing the iso-deformation field of the membrane (white dashed lines) during deformation allows the better appreciation of the presence of deformation gradients, as shown in Fig. 2f. By carefully characterizing the spatial variation of the strain magnitude in the membrane, we found that the deformation in the central region of the membrane (266 × 266 μm^2^) was relatively constant (±0.4 % variation in strain magnitude) and compares to other microfabricated stretching devices (Moraes et al. 2010, 2013; Kamotani et al. 2008). Typically, a pressure of 0.1 atm in the low pressure chambers induced a deformation of about 20 % in the center part of the membrane and is highly consistent between devices. Six devices have been used to quantify the repeatability of the fabrication process. At most, we observed a variation in the strain magnitude of ±2.6 % at 22.5 % deformation between devices, as depicted in Fig. 2g, h. We also investigated the repeatability of the strain field over time and found very little change in the magnitude of the deformation over 20 h under constant low pressure conditions, as depicted in Supplementary Fig. 3. Exploiting the ability to independently control the deformation along each orthogonal axis allowed us to expose cells to horizontal or vertical uniaxial strain fields. The simple relationship between pressure in the low pressure chambers and membrane strain allowed us to easily interpolate and precisely induce the desired strain magnitude along both axes.

### Cellular responses to dynamic and complex strain fields

**Fig. 3:**
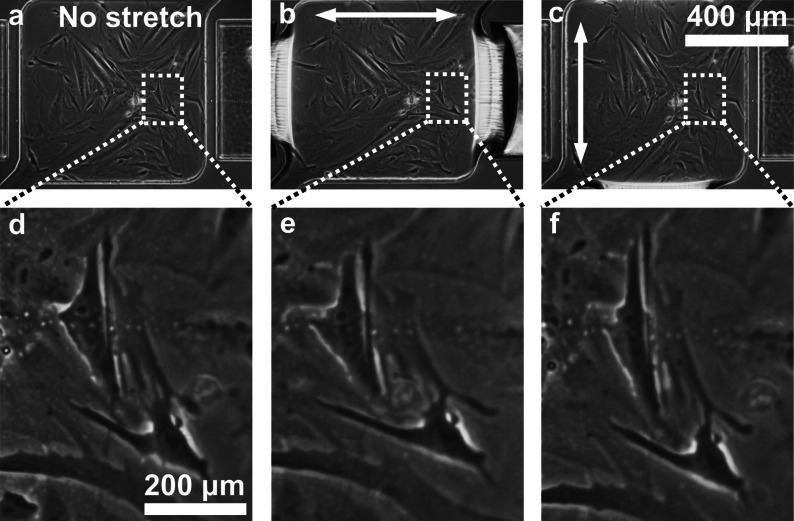
**a**–**c** Phase-contrast images of the same group of cells immobilized to the suspended membrane exposed at first to no deformation (**a**) and then exposed to a *horizontal* (**b**) and *vertical* deformation (c). *Arrows* indicate stretching directions. **d**–**e** *Insets* showing a particular group of cells exposed to the corresponding strain fields

Figure 3 demonstrates the device’s ability to apply complex strain fields, by inducing a deformation along two orthogonal directions. Figure 3a shows a phase-contrast image of the cells prior to deformation. HFF cells, immobilized on the suspended membrane, were then stretched, subject to a uniaxial strain of a magnitude of 20 % along the horizontal and vertical directions, as highlighted in Fig. 3b, c, respectively. Figure 3d, e are insets showing the instantaneous change in cell morphology during substrate stretching for the selected group of cells.

**Fig. 4:**
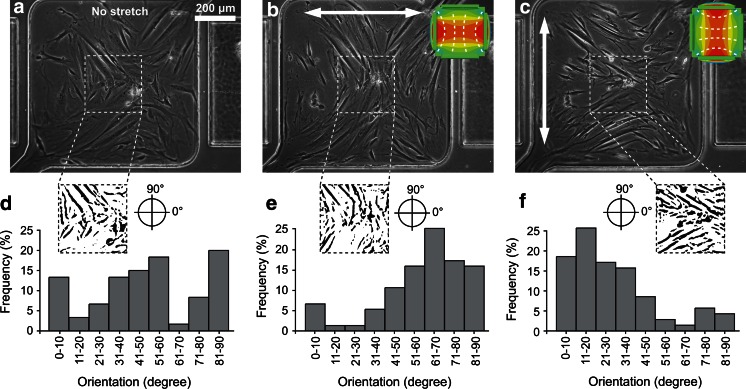
**a** Cells cultured for 24 h in the device prior to perform the cyclic stretching experiment. **b** Cells exposed to a sinusoidal cyclic deformation along the *horizontal* direction with an amplitude of 20 % and a frequency of 0.5 Hz for 8 h. **c** Same group of cells exposed to the same strain field but this time along the *vertical* direction for 16 h. *Insets* in **b** and **c** reveal the contour map of the magnitude of the membrane deformation (finite element simulation; see Online Resource 1), and the *dotted white lines* highlight the transversal contours. The cells align to follow these lines as well. **d** Cells are randomly orientated before inducing deformation. **e** Cells are mostly aligned along the *vertical* direction after 8 h of uniaxial stretching along the *horizontal* direction. **f** Cells are mostly aligned along the *horizontal* direction after 16 h of stretching along the *vertical* directions

The HFF cells of Fig. 4a are first exposed to a cyclic uniaxial strain field (20 % in magnitude; 0.5 Hz) along the horizontal direction, inducing a collective alignment of the cells along the vertical direction after 8 h, as highlighted in Fig. 4b. Then the orientation of the strain field is suddenly changed to mechanically stimulate the same cells along the vertical direction with the same magnitude and frequency as before. This induces a collective re-alignment of the cells along the horizontal direction after 16 h. As revealed in Fig. 4c, the cells have completely reoriented themselves horizontally as they align perpendicularly to the stretching direction, in agreement with previous work (Wang et al. 2001; Jungbauer et al. 2008). Cellular orientation under different conditions was quantified as shown in Fig. 4d–f. These histograms show the absolute value of the angle the cells assume with respect to the horizontal. Cells are randomly oriented before imposing deformation, as depicted in Fig. 4d. Reorientation occurs as the number of features orientated along the vertical (Fig. 4e) and horizontal (Fig. 4f) axes increases following a cyclic uniaxial mechanical deformation of the cells along the horizontal and vertical axes respectively.

**Fig. 5:**
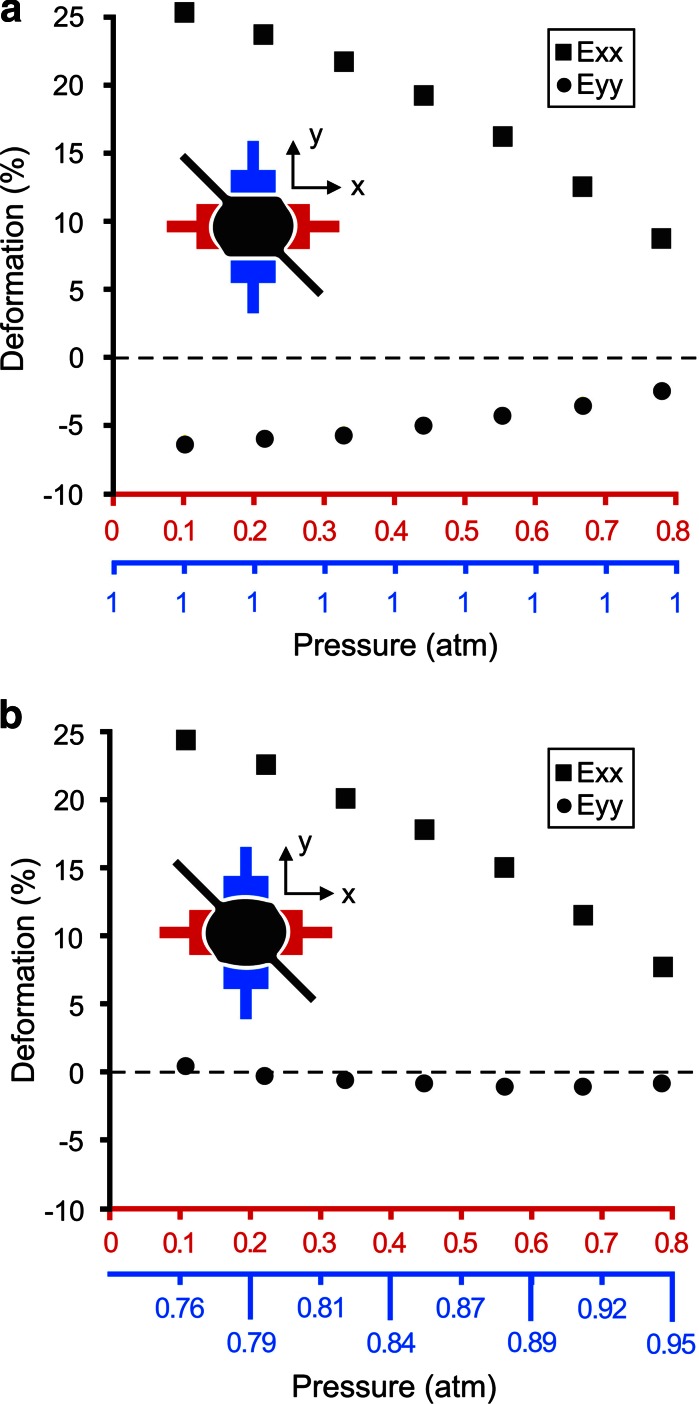
**a** Deformation-pressure relationship for a standard uniaxial strain field where the principal deformation occurs along the *horizontal* direction (scale and low pressure chambers colored in *red*) with the presence of a compressive strain along the *vertical* direction. Note that the low pressure chambers, along the *vertical* direction, are *left* at atmospheric pressure (scale and low pressure chambers colored in *blue*). **b** Deformation-pressure relationship for a pure uniaxial strain field where the principal deformation occurs along the *horizontal* direction while applying a stretch along the *vertical* direction to eliminate any compressive strains

When stretching the membrane in one direction, the suspended membrane contracts in the orthogonal direction, as expected. The data shown in Fig. 2g, h reveal this orthogonal compression. However, this effect can be minimized by compensating the compression by simultaneously stretching the membrane in the direction orthogonal to the main axis of stretching. This is illustrated in Fig. 5 where a uniaxial strain field is applied in the x-direction, while the compression in the y-direction is suppressed by simultaneously stretching in the perpendicular direction. The ability to induce strains using four independent low pressure chambers is unique in that it gives more control over the membrane’s strain field.

## Discussion

The recent development of microscale stretching devices has provided numerous insights into the kinetics of cellular responses to mechanical strain, at various time scales (milliseconds to hours) (Huh et al. 2010; Huang and Nguyen 2013; Kim et al. 2012; Moraes et al. 2010; Jungbauer et al. 2008; Moraes et al. 2013; Wang et al. 2001; Chen et al. 2013). Here, we build upon existing designs and present a microfabricated device that allows cells to be exposed to a strain field that can be controlled in two orthogonal directions independently. Existing approaches typically employ only uniaxial strain and do not possess the ability to dynamically change its direction. This provides the ability to change strain directions on the fly and also to create dynamic, complex and anisotropic strain fields. This approach provides a method for studying cellular reorientation resulting from complex and dynamic strains that better mimic what happens in vivo. Similarly, the work of Moraes et al. (2010, 2013) demonstrated a device that could independently change the radial and circumferential strain components, albeit with a maximum strain magnitude of 6 %. Our device is able to independently change both of the strain-field components dynamically with a maximum strain magnitude of 20 %. Importantly, the device allows the investigation of the effects of pure uniaxial or standard uniaxial stretching on cellular responses. Indeed, the effect of deforming cells perpendicularly to their orientation axis can induce severe disruption of microarchitecture of valve endothelial cells (Balachandran et al. 2011). Consequently, precise control of the strain field (pure uniaxial, standard uniaxial, biaxial, equibiaxial) as well as its direction and magnitude, will facilitate a systematic understanding of how cells respond to the complex, anisotropic and time-varying strain fields they encounter in vivo.

HFF cells respond to cyclic substrate deformations by changing their morphology and orientation. Indeed, cells undergo morphological changes under uniaxial stretching by orienting themselves almost perpendicularly to the stretching direction. The orientation of the cells reflects the slight non-uniformity of the applied strain field, as evident from the inset in Fig. 4b, c. As revealed by the strain maps obtained experimentally and from finite element simulations (Fig. 2d, f), the magnitude of the vertical deformation of the membrane is non-uniform and follows a curved shape, the gradient of which is estimated by the white dashed lines. This arrangement suggests that individual cells are sensitive to local strain variation. It is still not clear what mechanisms are responsible for this behavior found in many cell types, but it is hypothesized that cells position themselves to experience the least amount of deformation (Wang et al. 2001; Faust et al. 2011). To our knowledge, cell response to non-uniform strain fields has never been investigated before. Given the microscale dimensions of our device, it is possible to investigate the effect of strain field gradients across the same cell while monitoring cellular remodeling and migration. In other applications, up-sizing the device dimensions would provide for larger areas with uniform strain, where a greater number of cells could be exposed to similar deformations.

The integration of independent biaxial stretching capabilities on a microfluidic device provides precise control over the biochemical and mechanical environments experienced by cells. We have demonstrated that cells are able to proliferate in the device and reorient themselves in response to applied strain. The ability to induce deformation along two orthogonal directions allows the investigation of how anisotropic strain modulates the mechanisms governing cellular proliferation, organization and cytoskeletal remodeling in response to cyclic stretch (Goldyn et al. 2009; Chen et al. 2013; Jaalouk and Lammerding 2009). This may contribute to our understanding of how complex and anisotropic mechanical forces and strain originating in the extra-cellular matrix couple to the cytoarchitecture. Building upon the designs of previous microfluidic or macroscale stretching devices, we present an approach that provides the user with a unique ability to generate changing, anisotropic and time-varying strain fields in order to more closely mimic the complexities of strains occurring in vivo.

## Electronic supplementary material

Below is the link to the electronic supplementary material.

Supplementary material 1 (PDF 70 kb)

Supplementary material 2 (MP4 428 kb)

Supplementary material 3 (MP4 422 kb)

Supplementary material 4 (PDF 1603 kb)
