# Supplementary material for: A microscale anisotropic biaxial cell stretching device for applications in mechanobiology
Source: Biotechnol Lett. 2013 Oct 16;36(3):657–65. doi: 10.1007/s10529-013-1381-5 (PMC3964308; doi:10.1007/s10529-013-1381-5)
Supplement: Supplementary file 1 — Supplementary material 1 (PDF 70 kb) [file 10529_2013_1381_MOESM1_ESM.pdf]

**Electronic Supplementary Material**

# A microscale anisotropic biaxial cell stretching device for applications in mechanobiology

Biotechnology Letters

Dominique Tremblay[1], Sophie Chagnon-Lessard[1], Maryam Mirzaei[1], Andrew E. Pelling[1,3,4] and Michel Godin[1,2,*]

[1]Department of Physics, University of Ottawa, 150 Louis-Pasteur, Ottawa, ON, Canada, K1N 6N5

[2]Ottawa-Carleton Institute for Biomedical Engineering, University of Ottawa, 161 Louis Pasteur, Ottawa, ON, Canada, K1N 6N5

[3]Department of Biology, University of Ottawa, 30 Marie Curie, Ottawa, ON, Canada, K1N 6N5

[4]Institute for Science, Society and Policy, University of Ottawa, 55 Laurier Ave. East, 10th Floor, Ottawa, ON, Canada, K1N 6N5

*Correspondence and request for materials should be addressed to:

Michel Godin

Department of Physics

University of Ottawa

150 Louis-Pasteur

Ottawa, ON

Canada, K1N 6N5

Tel.: 1-613-562-5800 x6771

Fax: 1-613-562-5190

email: michel.godin@uottawa.ca

## Device fabrication and assembly

To fabricate the 3-layer PDMS device, two master molds were prepared by photolithography on polished silicon wafers with SU-8 2050 negative photoresist (Microchem, Newton, MA, USA). Masters were further exposed to Trichloro(1H,1H,2H,2H-perfluorooctyl)silane vapor (Sigma-Aldrich, USA) to ease PDMS removal after curing. The elastic properties of the PDMS were controlled by varying the ratio of curing agent relative to the base monomer. The bottom and top sections of the device used a ratio of 1:15 and was cured for 2 h at 80 °C, whereas the membrane had a ratio of 1:20 to increase the elasticity and was cured for 1 h at 80 °C. Fluorescent beads (FluoSpheres, 200 nm, Invitrogen, CA, USA) were embedded into the membrane to act as stationary markers in order to calibrate the membrane deformation during stretching. Initially suspended in an aqueous solution, the fluorescent beads were resuspended in isopropanol at a ratio of 1:10 to enhance mixing with the PDMS. PDMS (1:20) with embedded beads was spin-coated on a clean silicon wafer coated with the releasing agent at a speed of 3000 rpm for 5 min. The bottom section and the membrane were air-plasma treated for 30 s at 30 W, bonded together and further cured for 30 min at 80 °C. The bottom section, which is now bonded to the membrane, was peeled off from the underlying wafer. The membrane was then punctured manually with a needle in line with the low pressure and the fluidic channel ends. Holes were punched in the top section using a biopsy punch, in order to accommodate external tubing. The bottom (with membrane) and top sections were again air-plasma treated. The top section was carefully aligned with the bottom part using a mask aligner (OAI, CA, USA). The whole assembly was further cured for 30 min at 80 °C.

## Incubation chamber

Since PDMS is porous to gas, the device is kept at 37 °C in a humid atmosphere of 5% $CO_2$ and 95% air to maintain culture medium pH at a physiological value. As depicted in Supplementary Fig. 1 in Online Resource 4, the device in placed in a petri dish, which is put into a petri dish heater, and kept at a temperature on 37 °C. A lid with holes allows the insertion of external tubing and gas input of 5% $CO_2$ and 95% air at a flow rate of 100 ml/min. To maintain humid conditions, sponges filled with water are placed inside the petri dish. The whole set up is covered with a plexi-glass heating box to prevent condensation from forming on the lid covering the petri dish heater, and to keep the lid temperature at 38 °C.
