# Supplementary material for: A microscale anisotropic biaxial cell stretching device for applications in mechanobiology
Source: Biotechnol Lett. 2013 Oct 16;36(3):657–65. doi: 10.1007/s10529-013-1381-5 (PMC3964308; doi:10.1007/s10529-013-1381-5)
Supplement: Supplementary file 4 — Supplementary material 4 (PDF 1603 kb) [file 10529_2013_1381_MOESM4_ESM.pdf]

Supplementary Figures

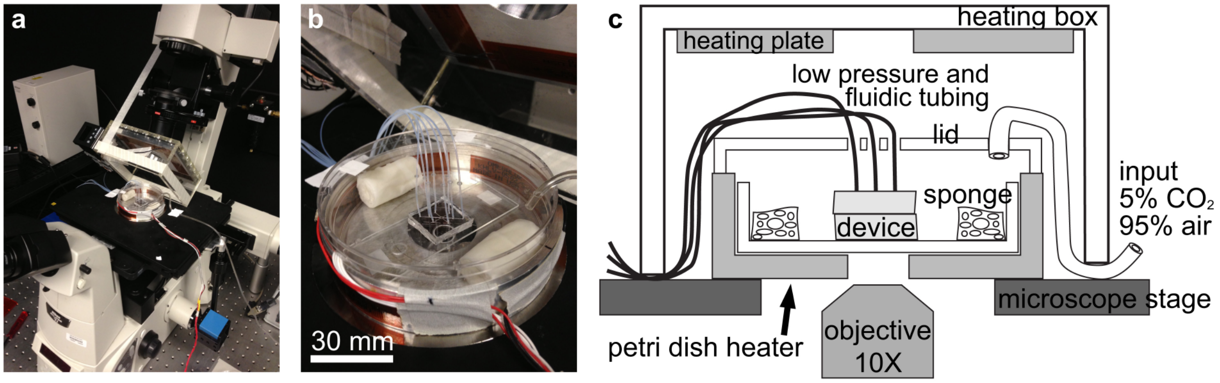

Supplementary Figure 1 a-b) Photograph of the incubation chamber on the microscope stage that allows the culture of cells for extended periods of time. c) Schematic of the incubation chamber showing details of the assemblage.

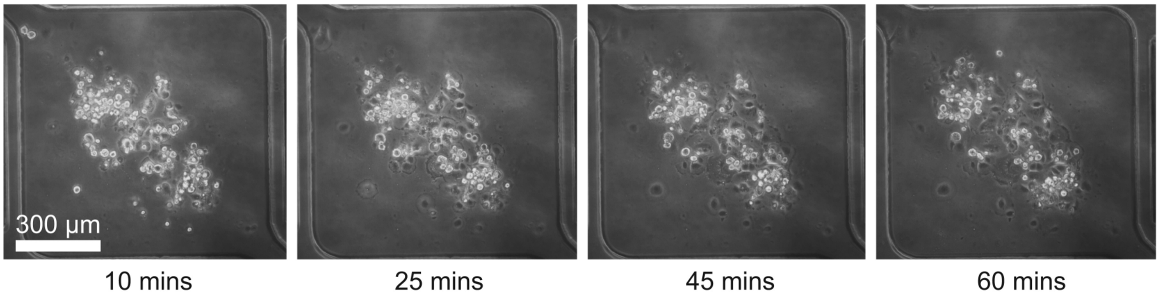

Supplementary Figure 2 Sequence of phase contrast images illustrating the attachment of the cells onto the membrane in the cell chamber during the seeding procedure.

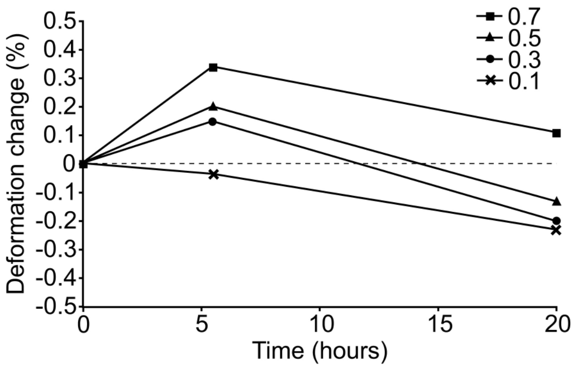

Supplementary Figure 3 Variations of the maximum deformation induced in the membrane over a period of time of 20 hours. The membrane was exposed to a cyclic deformation by applying a pressure of 0.7, 0.5, 0.3 and 0.1 atm in the low pressure chambers at a frequency of 0.5 Hz.

Supplementary Videos

Supplementary Video ESM_2 and ESM_3: The symmetry of the device allows to perform live-cell imaging as the membrane on which cells are attached stays in the focal plane of the objective during cell stretching. They show cells under cyclic stretching during one cycle along the horizontal (ESM_2) and vertical direction (ESM_3).
